# Photodynamic Therapy Using a Heavy-Atom-Free G‑Quadruplex-Targeted Photosensitizer to Efficiently Regress Rhabdomyosarcoma Tumors In Vivo

**DOI:** 10.1021/acsptsci.5c00061

**Published:** 2025-04-16

**Authors:** Eva Rodriguez-Marquez, Hanna Nord, Darío Puchán Sánchez, Ahmad Kassem, José María Andrés Castán, Marco Deiana, Clement Cabanetos, Nasim Sabouri, Jonas von Hofsten

**Affiliations:** † Department of Medical and Translational Biology, Umeå University, 90187 Umeå, Sweden; ‡ University Angers, CNRS, MOLTECH-ANJOU, SFR MATRIX, F-49000 Angers, France; § Institute of Advanced Materials, Faculty of Chemistry, 49567 Wrocław University of Science and Technology, 50-370 Wrocław, Poland; ∥ Department of Medical Biochemistry and Biophysics, Umeå University, 90187 Umeå, Sweden

**Keywords:** photodynamic therapy, G-quadruplex, photosensitizer, rhabdomyosarcoma, cancer, zebrafish

## Abstract

Rhabdomyosarcoma is a highly aggressive soft tissue cancer that predominantly affects children and adolescents. Current treatment outcomes are poor, highlighting the urgent need for potent therapeutic alternatives. Preclinical research on photodynamic therapy (PDT) continues to gain attention as a promising and minimally invasive treatment strategy. Recently, PDT using the heavy-atom-free photosensitizer dibenzothioxanthene imide (**DBI**), which targets cancer-associated G-quadruplex (G4) DNA, has demonstrated high efficacy at nanomolar concentrations. In here, transgenic zebrafish with rhabdomyosarcoma tumors were utilized to evaluate the therapeutic potential of **DBI** treatment. We demonstrate that photoactivated **DBI** efficiently induce localized tumor necrosis, resulting in significant rhabdomyosarcoma regression compared to untreated controls. In fact, in comparison to the healthy cells surrounding the tumor, a high level of G4s was detected, as visualized by a G4-specific antibody. Notably, muscle and nerve cells within the treated tumor area were particularly affected, further underscoring its potency. These findings position **DBI** as a promising candidate for PDT in the treatment of rhabdomyosarcoma, offering selective G4-targeting capabilities and delivering robust therapeutic outcomes in in vivo models.

## Graphical abstract

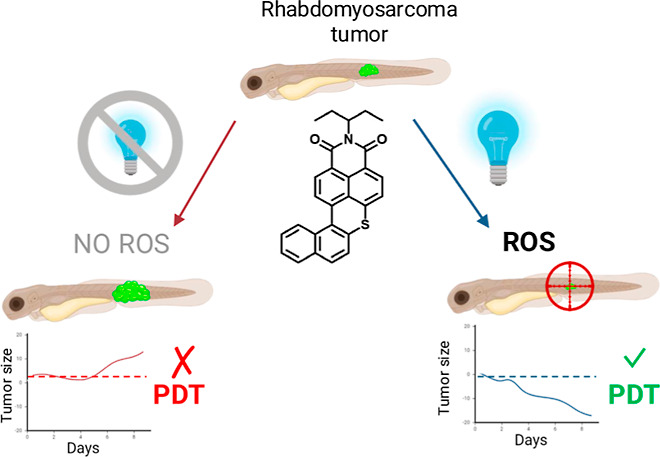

Photodynamic therapy (PDT) involves the administration, selective accumulation, and subsequent photoactivation of drugs, known as photosensitizers (PS), to induce cell death in cancerous cells. Upon photoactivation by a specific wavelength of light, the PS absorbs the photon energy and undergoes a transition from its ground state to an excited singlet state. This energy is then transferred to molecular oxygen present in the surrounding tissue through intersystem crossing to the PS’s triplet state, inducing selective tissue destruction through the localized generation of reactive oxygen species (ROS). These short-lived, highly reactive molecules primarily damage cells that accumulate the PS, resulting in targeted destruction of cancer tissue. This makes PDT a minimally invasive alternative to traditional cancer therapies. A key advantage of PDT is its precision, allowing for the elimination of diseased cells by initiating different cell death pathways, such as apoptosis and necrosis, as well as damaging the microvasculature and inducing local inflammatory reactions in targeted tissues.^1,2^

PDT has proven effective in treating various cancers, including nonmelanoma skin cancer,^3^ head and neck cancers,^4^ esophageal cancer,^5^ and lung cancer.^6^ While these cancers have been extensively studied, there is growing interest in exploring PDT’s potential for treating rhabdomyosarcoma (RMS), a highly aggressive pediatric soft tissue cancer. Notably, early studies have demonstrated the success of PDT in vitro with hypericin-based approaches showing promise against rhabdomyosarcoma cells, paving the way for further exploration of this promising treatment in RMS.^7^ More recent research has revealed a novel approach combining PDT and radiotherapy for selectively targeting RMS cells in vitro.^8^ However, despite these advances, one major challenge remains: translating these findings into in vivo settings, which is crucial for advancing into clinical application.

RMS originates from skeletal muscle progenitors and is the most common pediatric soft tissue sarcoma, and the third most common extracranial solid tumor in childhood. It accounts for approximately 3–5% of all pediatric cancers with an annual incidence of about 4.5 cases per million children under 15 years of age.^9^ Current treatment for RMS involves a multimodal approach, including surgery, radiation therapy, and intensive chemotherapy. However, the outcome for children with metastatic disease is very poor, with overall survival rates at 3 years of 25–30%.^10^ The significant morbidity and mortality associated with RMS highlight the urgent need for innovative, more effective therapeutic strategies, including approaches such as PDT.

Advances in light delivery and PS design have improved PDT precision.^11^ However, conventional PSs often rely on heavy-atoms or complex and synthetically demanding polyaromatic structures, leading to challenges such as dark toxicity, high costs, and synthesis difficulties.^12^ These issues highlight the need for cost-effective, easily synthesized, heavy-atom-free PSs for clinical use. Recently, we have reported several fluorescent small heavy-atom-free PSs that incorporate a twisted π-conjugated system with an unusually high quantum (ΦΔ = 0.95) for a small molecule, exceptional phototoxic efficiency, and negligible in dark toxicity.^13–15^ One of the PS, namely dibenzothioxanthene imide (**DBI**), prepared in only two synthetic steps, recently demonstrated phototoxic effect at nanomolar concentration by targeting G-quadruplex (G4) DNA structures.^14^

G4s are four-stranded DNA architectures that arise in guanine-rich regions. These structures are stabilized by Hoogsteen hydrogen bonding between stacked G-quartets and by coordination with monovalent cations, such as potassium (K^+^). G4 structures typically form when guanine-rich sequences become transiently single-stranded during processes such as replication, transcription, or DNA repair. Because G4s can interfere with fundamental DNA processes and contribute to genomic instability, they have gained attention as promising therapeutic targets—particularly in cancer. In addition, tumor cells often exhibit elevated G4 formation compared to healthy cells,^16,17^ making them more vulnerable to G4-targeted approaches using small-molecule ligands. As a result, G4s have emerged as compelling and innovative targets for cancer therapy.^18–24^

Additionally, guanine-rich sequences are particularly susceptible to oxidative damage caused by ROS, leading to the formation of oxidized guanine bases.^25,26^ In fact, **DBI** efficiently generate ROS and induces oxidatively damaged guanine bases and double-stranded DNA breaks in cancer cell lines, tumor organoids, and 2 day postfertilization (dpf) zebrafish embryos.^14^ These findings highlight **DBI** as a promising candidate for further investigation in cancer therapy.

Zebrafish have become a prominent model organism in cancer therapy due to their transparent embryos, high genetic homology with humans,^27^ and the ability to visualize tumor progression in vivo.^28^ In zebrafish, over 100,000 G4 sequences have been mapped using the G4-sequencing method and their roles as transcriptional cis-regulatory elements have been investigated.^29–31^ Zebrafish models are particularly valuable for studying the pathogenesis of cancers like RMS.^32^ For example, overactivation of the RAS signaling pathway in zebrafish induces fusion-negative RMS, closely mimicking key aspects of the human disease.^33^ Additionally, zebrafish’s aquatic environment facillitates efficient pharmacological testing, as compounds can be dissolved in dimethyl sulfoxide (DMSO), a low-toxicity solvent that enhances membrane permeability without causing damage and is easily absorbed by embryos.^34^

In this study, we evaluated the feasibility of using **DBI** for treating RMS tumors in a zebrafish model. Upon photoactivation, **DBI** effectively triggered localized tumor apoptosis, resulting in substantial tumor regression when compared to untreated controls, highlighting **DBI** as a compelling candidate for PDT in the treatment of RMS.

## Results

**DBI** was synthesized following a more direct and efficient procedure based on our previously published study^14^ (Figure 1A). To evaluate the uptake and activation of the photosensitizer **DBI** in zebrafish larvae, wildtype zebrafish were incubated with either **DBI** or DMSO as control (Figure 1B).

**Figure 1:**
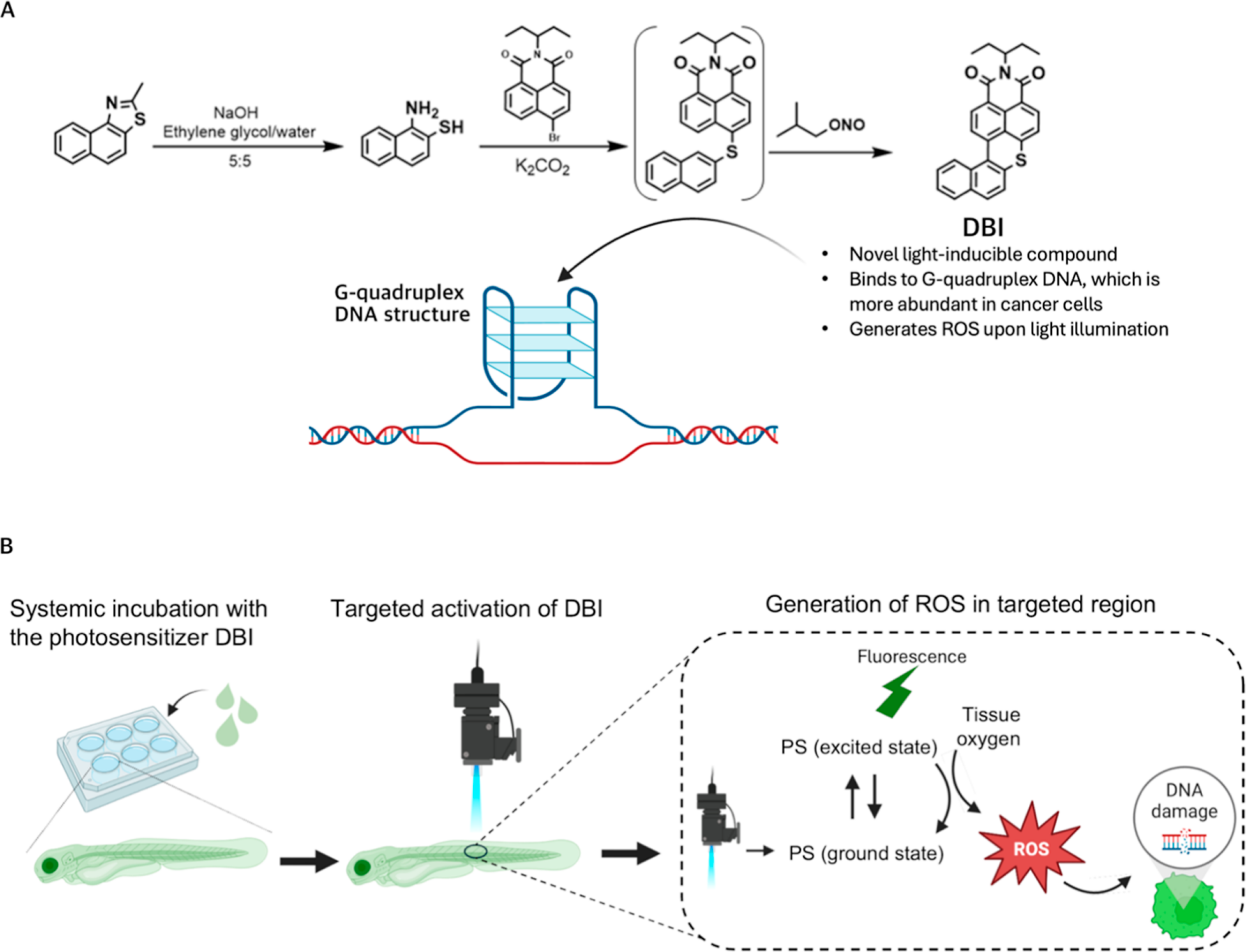
**DBI**-mediated PDT. (A) Synthesis of **DBI**. (B) Schematic illustration of PDT using **DBI**. Zebrafish are systemically incubated with **DBI**, which is subsequently activated in the target region. Upon activation, **DBI** releases fluorescence and generates ROS, leading to DNA damage and apoptosis.

At 2 dpf, both DMSO and **DBI**-treated larvae appeared morphologically similar under grayscale imaging. However, upon blue LED-light photo irradiation, only the **DBI**-treated larvae displayed a significantly increased green fluorescent signal, indicating successful photosensitizer uptake (Figure 2A). Similar results were observed at 7 dpf, confirming that the zebrafish larvae retain the ability to uptake **DBI** from the growth media as they develop (Figure 2A).

**Figure 2:**
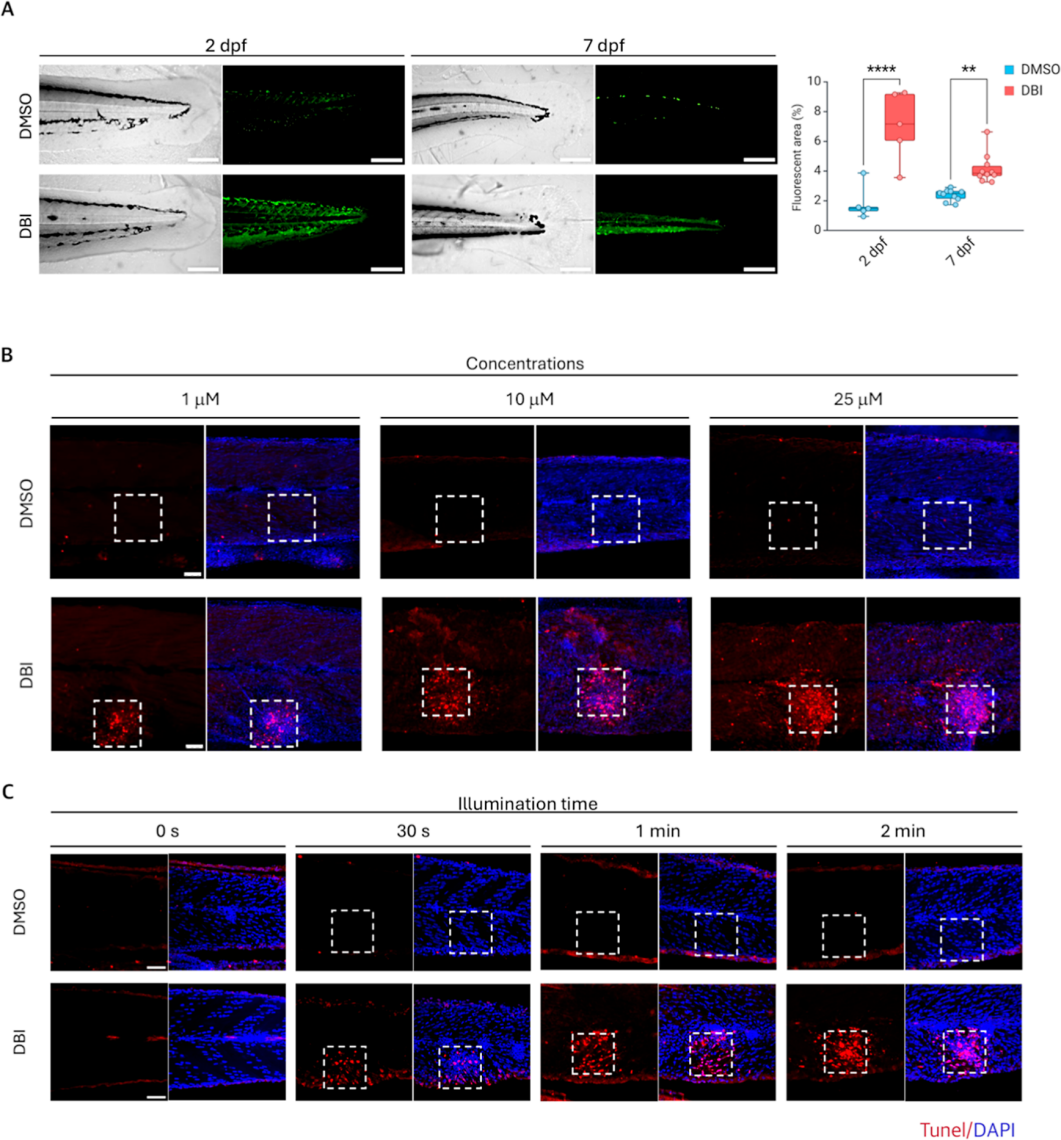
Uptake and activation of **DBI** in zebrafish. (A) Confocal laser scanning microscopy images of 2 days postfertilization (dpf) and 7 dpf AB zebrafish larvae treated with DMSO (control) or **DBI**, followed by illumination. Grayscale images (left) and corresponding green fluorescent signals (right) are shown. Scale bar = 200 μm. *p* < 0.01 (**), *p* < 0.0001 (****). (B,C) Apoptosis detection in zebrafish after DMSO or **DBI** treatment, followed by illumination, using different **DBI** concentrations: 1,10 and 25 μM (B) and different light exposure durations: 0 s to 2 min (C). Dashed squares indicate the illuminated areas. Apoptotic cells were stained with TUNEL (red), and nuclei were counterstained with DAPI (blue). Scale bar = 50 μm.

To assess the cytotoxicity of **DBI** in vivo, zebrafish larvae were incubated with either **DBI** or DMSO and subjected to PDT with blue light. Different **DBI** concentrations (Figure 2B) and light exposure durations (Figure 2C) were tested. Cytotoxicity was evaluated by detecting apoptotic DNA fragmentation using TUNEL staining. Apoptosis was observed only in **DBI**-treated embryos exposed to blue light, with higher concentrations (Figure 2B) and longer light exposure (Figure 2C) leading to increased apoptosis. In contrast, no TUNEL staining was detected in the DMSO-treated controls (Figure 2B,C) or in the absence of light exposure (Figure 2C). These results confirm that PDT-induced apoptosis requires both the presence of the photosensitizer and light exposure, indicating its potential for targeted cancer treatment.

Next, we evaluated the efficacy of **DBI** in treating RMS in a zebrafish model. RMS tumors were induced in the TgBAC­(pax3a:EGFP)^i150^ transgenic zebrafish line by microinjecting rag2-kRASG12D, a mutated form of kRAS specifically expressed in muscle progenitors, leading to the development of RMS (Figure 3A). Once tumors were detectable, they were imaged using a confocal microscope, and the fish were incubated with **DBI**. **DBI** was then activated by blue light exposure by specifically localizing the light to the tumor region. Tumor response to **DBI** treatment was assessed the following day through imaging (Figure 3A). Control groups included DMSO treatment, with or without light exposure, as well as **DBI**-treatment without blue light activation. RMS tumors were identified by the abnormal accumulation of pax3a:EGFP, as previously described^35^ (Figure 3B). Zebrafish with photoactivated **DBI** showed a marked reduction in tumor size 4 days post treatment, while tumors in the DMSO-control group increased in size (Figure 3C). Quantitative analysis confirmed a significant reduction in tumor area at 4–5 days post treatment in the **DBI** + light group compared to all three control groups (**DBI** – light, DMSO + light, and DMSO – light) in pax3a:EGFP zebrafish with kRAS-induced RMS (Figure 3D,E). These results demonstrate that light-activated **DBI** is an effective treatment for RMS in the zebrafish model and imply its broad potential for tumor treatment.

**Figure 3:**
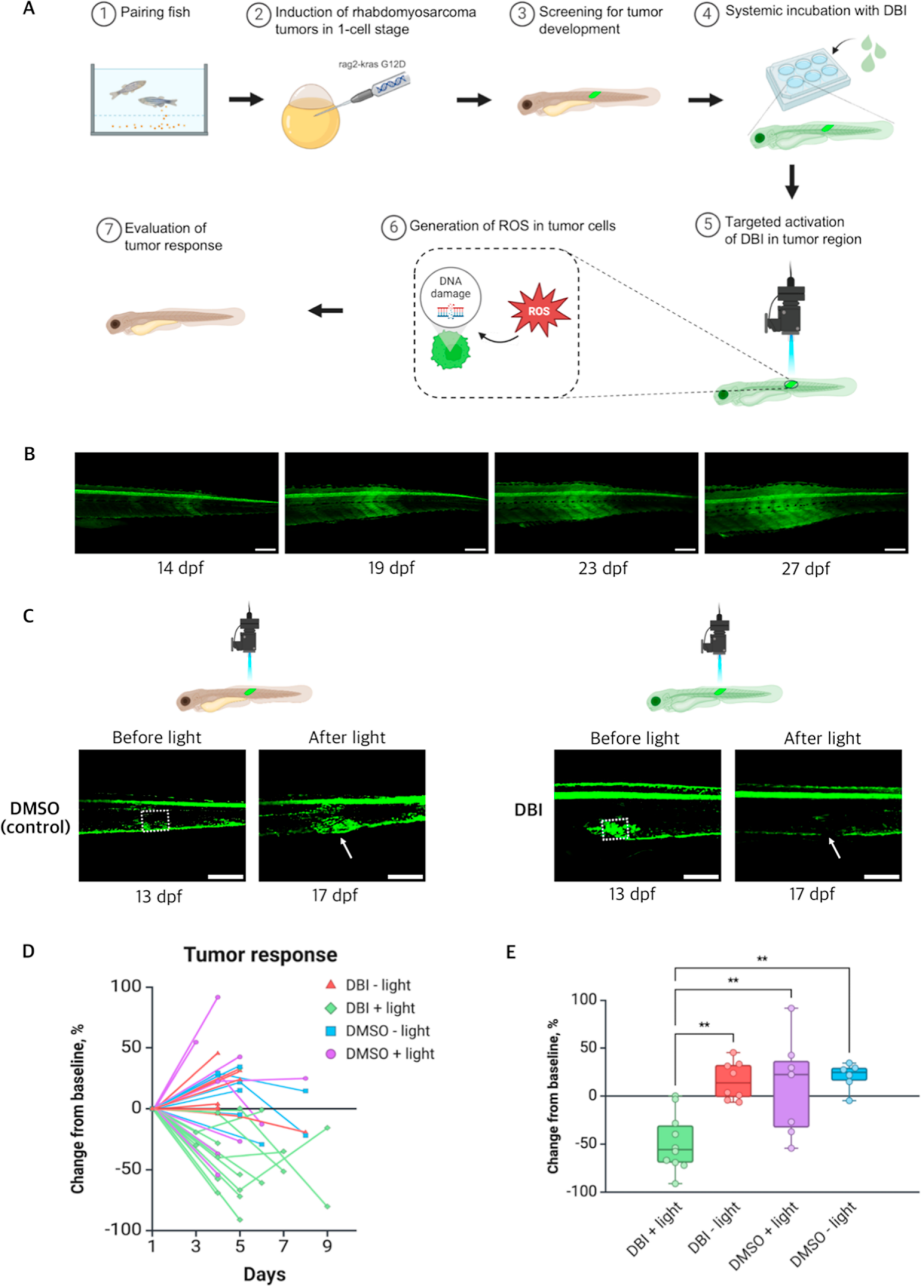
Effects of PDT on kRAS-induced RMS tumors in pax3a:EGFP zebrafish. (A) Schematic illustration of the induction of RMS tumors in zebrafish, followed by treatment with **DBI** and evaluation of the tumor response. (B) Representative image of RMS tumor progression in pax3a:EGFP zebrafish line, from 14 to 27 days post fertilization (dpf). The tumor region is identified by high levels of GFP expression. Scale bar = 200 μm. (C) Confocal images of tumors before and after treatment with DMSO + light (left) and **DBI** + light (right). Dashed squares indicate the illuminated areas. Tumor responses are indicated with the white arrows. Scale bar = 200 μm. (D) Comparison of tumor area, expressed as a percentage of initial size, after treatment across four conditions: **DBI** + light (treatment), and three controls: **DBI** – light, DMSO + light, and DMSO – light. (E) Percentage change in tumor area at 4–5 days post-treatment across the four conditions. *p* < 0.01 (**).

Photoactivated **DBI** efficiently reduced RMS progression (Figure 3C–E) and induced cell death in healthy embryonic muscle tissue (Figure 2B,C).

To minimize excess tissue damage, it is crucial to limit photoactivation to the treatment area. To assess whether RMS tumor cells were more sensitive to **DBI**, we compared heathy muscle tissue with RMS tumor tissue, using **DBI** with and without light-activation, followed by TUNEL analyses (Figure 4A,B). Comparisons of TUNEL staining in the illuminated areas showed significantly higher levels of apoptosis in RMS tumor tissue compared to healthy controls (Figure 4C).

**Figure 4:**
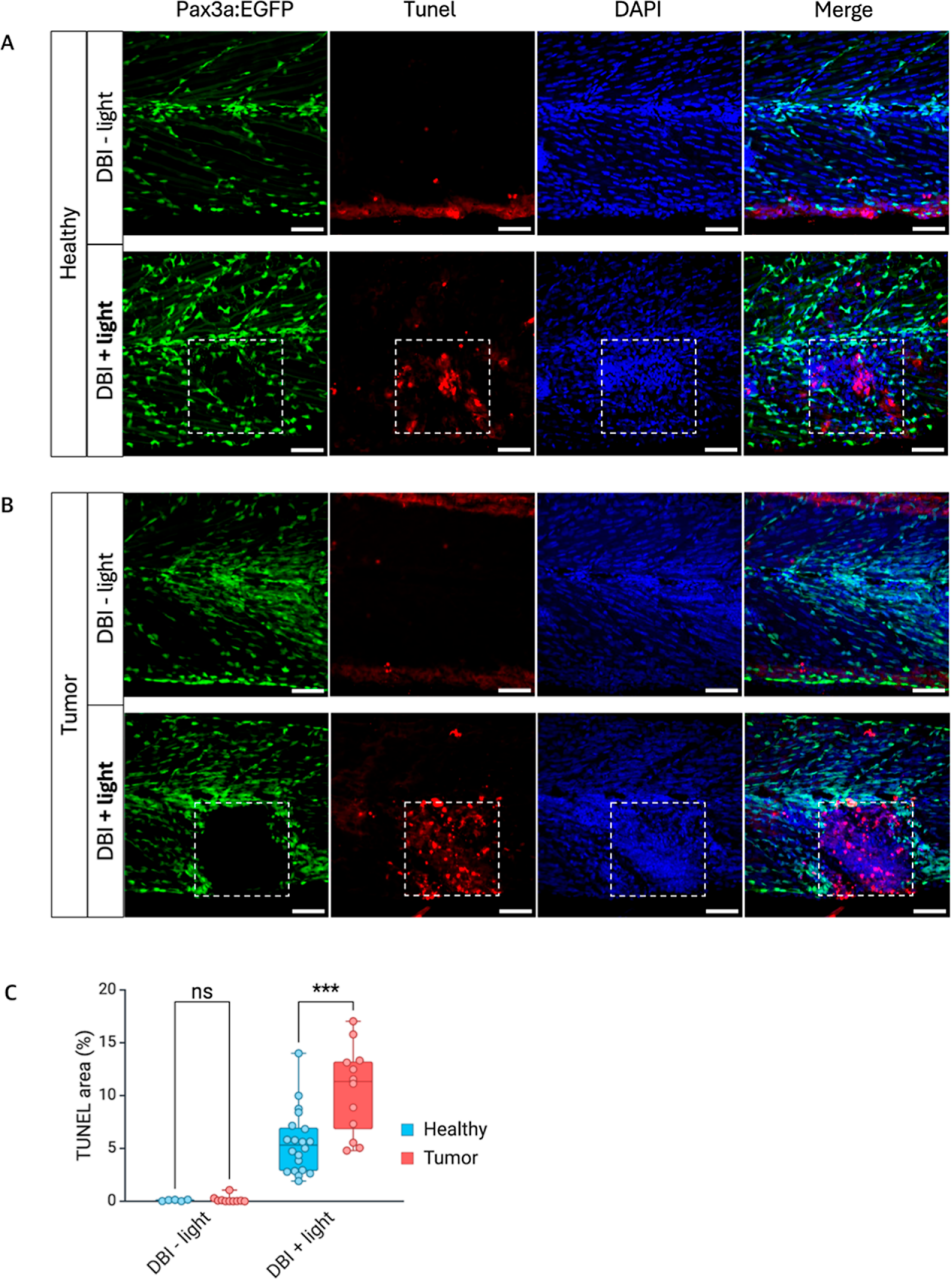
Apoptosis detection in zebrafish after **DBI** treatment. (A) Representative images of 2 week-old pax3a:EGFP zebrafish without tumors treated with **DBI**, followed or not by illumination. (B) Sibling zebrafish with rhabdomyosarcoma tumors treated with **DBI**, followed or not by illumination. Dashed squares indicate the illuminated areas. Apoptotic cells were detected with TUNEL staining (red), and nuclei were counterstained with DAPI (blue). Scale bar = 50 μm. (C) Quantification of TUNEL-stained area in healthy and tumor regions, treated with **DBI**, followed or not by illumination. *p* < 0.001­(***).

To assess whether G4 DNA contributes to the observed apoptotic effect in RMS (Figure 4B), we analyzed the RMS tumors for G4 content, using the BG4 antibody, which recognizes G4 structures in cells and tissues.^17,36^ Notably, a significant accumulation of BG4 signal was observed in the RMS tumors compared to the surrounding healthy region (Figure 5), suggesting that the enhanced sensitivity to **DBI** treatment in these tumors may be due to their elevated G4 levels.

**Figure 5:**
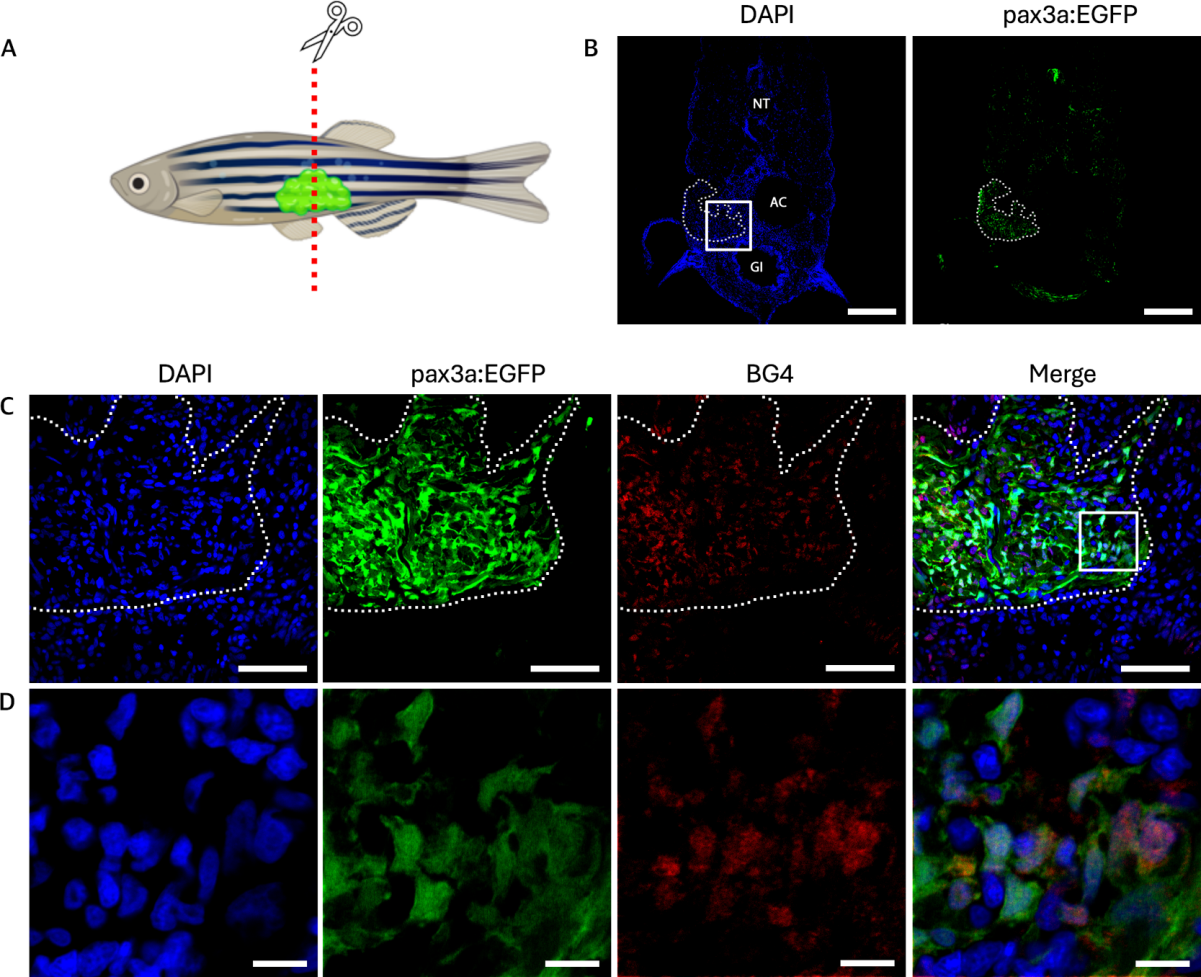
Elevated levels of G4 structures in RMS tissue sections of pax3a:EGFP zebrafish. (A) Schematic illustration of 2 month-old pax3a:EGFP zebrafish with RMS tumor. (B) Tissue section of the tumor, showing DAPI (left) and pax3a:EGFP (right). NT: neural tube, AC: abdominal cavity, GI: gastrointestinal tract. Dashed lines indicates where the tumor is located. Scale bar = 200 μm. (C) Zoom of the boxed region from (B), showing DAPI in blue, pax3a:EGFP expression in green, BG4 staining for G4 DNA in red, and a merged image of all channels. Scale bar = 50 μm. (D) Zoom of the boxed region from (C). Scale bar = 10 μm.

To further investigate, we evaluated cell proliferation in both healthy and RMS tissue using BrdU incorporation, which labels newly synthesized DNA during the S-phase of the cell cycle. Both healthy and RMS tissue showed similar levels of proliferation in the treated areas (Figure S1), arguing against an increased proliferation in RMS after disturbing the tumor by the **DBI** treatment. To further analyze how light-activated **DBI** affects different cell types and tissues associated with the RMS, Tg­(fli1a:EGFP; lyve1b:dsRED) zebrafish larvae that marks larger blood- and lymph-vessels were analyzed. Surprisingly, **DBI** treatment did not visibly damage the vessels marked by the transgenic insertions (Figure S2), suggesting that **DBI** has less pronounced toxic effect on the larger vessels in zebrafish larvae. However, areas treated with **DBI** showed significant damage when analyzed with a-bungarotoxin (α-btx) to mark neuromuscular junctions and acetylated tubulin to mark axons (Figure 6), indicating variable cell damage depending on the context.

**Figure 6:**
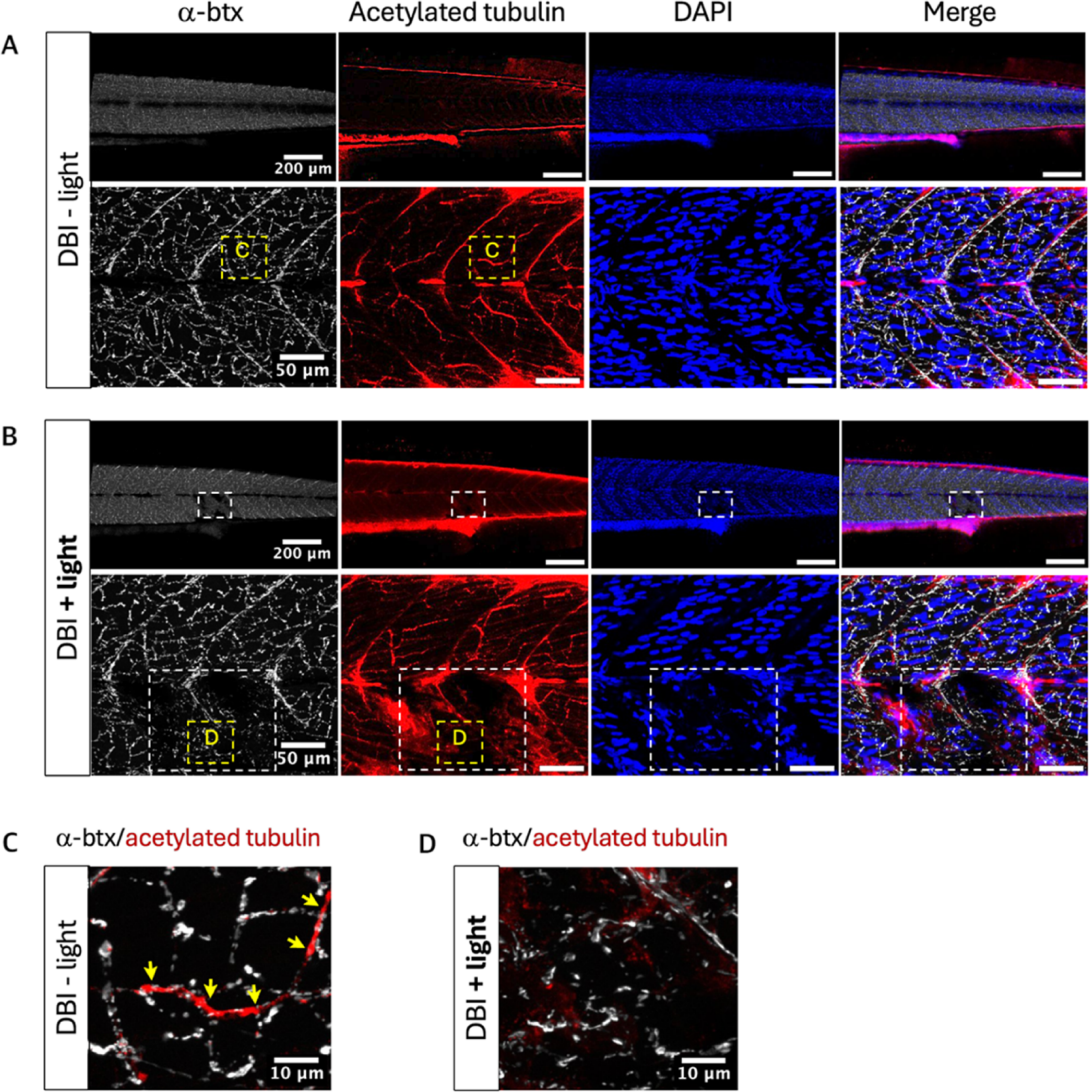
Effects of **DBI** on neuromuscular junctions. (A) Zebrafish treated with **DBI** without light. (B) Zebrafish treated with **DBI** and light. White dashed squares indicate the illuminated areas. (C) Zoom of a nonilluminated region from Figure 4A, taken from the yellow dashed square region. The yellow arrows indicate the overlap of α-btx and acetylated tubulin. (D) Zoom of the illuminated region from Figure 4B, taken from the yellow dashed square region. Neuromuscular junctions are shown by α-btx staining (white) and axonic microtubules are shown by acetylated tubulin staining (red). Nuclei were counterstained with DAPI (blue).

## Materials and Methods

### Synthesis of **DBI**

**DBI** was adapted from the previously reported method.^14^ First, a solution of 2 methylnaphtho­[1,2-*d*]­thiazole (2.5 g, 12.55 mmol) in a mixture of ethylene glycol and aqueous 50% NaOH (v/v = 5/5,30 mL) was refluxed under argon for 16 h before being poured into an ice–water bath and acidified to pH = 4 with 1 M HCl solution (Figure 1A). The organic phase was then extracted with diethyl ether, dried over MgSO4 and concentrated under reduced pressure. Used without further purification, the resulting 1-aminonaphthalene-2-thiol (460 mg, 2.62 mmol, 1.2 equiv) was added to a solution of 6-bromo-2 (pentan-3 yl)-1*H*benzo­[*de*]­isoquinoline-1,3­(2*H*)-dione (758 mg, 2.19 mmol, 1 equiv), and K_2_CO_3_ (166 mg, 1.2 mmol, 0.55 equiv) in 2-ethoxyethanol (30 mL) before being refluxed for 15 h. Then, isoamyl nitrite (0.775 mL, 6.56 mmol, 3 equiv) was added and the reaction mixture was subsequently refluxed for 5 extra hours. The mixture was cooled down before being diluted with DCM. The organic phase was washed twice with water and once with brine, then dried over MgSO4 and finally filtered. After removing the solvent by rotary evaporation, the crude was subjected to a silica gel column chromatography using DCM as eluent to afford the target **DBI** as an orange powder (300 mg, 33%). This new synthesis route increased the yield almost 2-fold (from 18% to 33%) compared to our previous report.^14^ One H NMR (300 MHz, CDCl_3_): δ (ppm) 8.72–8.58 (m, 2H), 8.45–8.38 (m, 2H), 7.90–7.83 (m, 1H), 7.80 (d, *J* = 8.6 Hz, 1H), 7.59–7.50 (m, 3H), 7.39 (d, *J* = 8.6 Hz, 1H), 5.15–5.02 (m, 1H), 2.36–2.20 (m, 2H), 2.00–1.84 (m, 2H), 0.91 (t, *J* = 7.5 Hz, 6H). 13C NMR (75 MHz, CDCl_3_): δ (ppm) 139.1, 136.8, 134.0, 133.5, 131.6, 130.4, 130.4, 129.2, 129.1, 127.5, 126.7, 125.9, 125.7, 124.0, 123.1, 121.1, 120.0, 118.6, 57.5, 25.1, 11.5. HRMS (MALDI) *m*/*z* calcd for C27H21NO2S: 423.1288, found: 423.1293. Monocrystals were obtained by slow evaporation of a mixture of MeOH/chloroform. CCDC number: 2083069.

### Zebrafish Maintenance

Zebrafish (*Danio rerio*) used in this study were maintained in compliance with the standard procedures at the Umeå University Zebrafish Facility. All animal experiments were approved by the Regional Ethics Committee at the Court of Appeal of Northern Norrland, (Dnr A6-2020).

### **DBI** Uptake and Activation in Zebrafish

AB wildtype zebrafish were used to study the uptake and activation of the photosensitizer **DBI**. Both 1 day-postfertilization (dpf) and 5dpf zebrafish were incubated in absence of light at 28.5 °C with 10 μM **DBI** ^14^ in E3 embryo medium^37^ for 24 and 48 h, respectively. Following incubation, the fish were anesthetized using tricaine (MS-222, Sigma-Aldrich, USA) at a concentration of 160 mg/L, and placed under a Nikon A1 confocal microscope. They were then exposed to blue light for 5 min to activate the **DBI**.^14^ As a control, AB wildtype zebrafish at 1 dpf and 5 dpf were incubated with DMSO (0.2% v/v) and exposed to the same conditions. Imaging was performed using a Nikon A1 confocal microscope and percentage of fluorescent area was quantified using ImageJ.

### Local Photostimulation and Cytotoxicity of **DBI** In Vivo

To test the cytotoxicity of **DBI** in vivo, 1 month-old AB wildtype zebrafish were incubated with 1, 10, or 25 μM of **DBI**, or DMSO as a control, in E3 medium at 28.5 °C in the dark for 2 days. Next, zebrafish were photostimulated for 2 min, using a Nikon A1 confocal microscope with 5% of 488 nm laser activity.

To assess the effects of different light exposures, 1 week-old AB wildtype zebrafish larvae were incubated with 1 μM of **DBI**,^14^ or DMSO as a control, in E3 medium at 28.5 °C in the dark overnight. Next, zebrafish were photostimulated using a Nikon A1 confocal microscope with 20% of 488 nm laser activity in a specific region, covering 2 somites above the yolk sac extension, for varying durations, ranging from 0 s to 2 min.

In both cases, TUNEL assay was conducted to detect apoptotic DNA fragmentation.

### Detection of Apoptosis by TUNEL Assay

Following local photostimulation, fish were kept in the darkness for 4 h, and fixed overnight in 4% paraformaldehyde (PFA) in PBS at 4 °C. To detect apoptotic DNA fragmentation, the Click-iT Plus TUNEL Assay with Alexa Fluor 594 dye (Invitrogen, C10618, USA) was used following the manufacturer's instructions. Briefly, zebrafish larvae were washed in PBS, permeabilized in cold acetone for 2 h at −20 °C, and washed in PBS. Samples were incubated with Tdt reaction buffer for 10 min at 37 °C, followed by incubation with Tdt buffer, EdTTP, and Tdt enzyme for 3 h at 37 °C. Afterward, the samples were washed with 3% BSA + 0.1% Triton X-100 in PBS for 5 min, and then PBS for 5 min. The Click-iT reaction cocktail, including SuperMix and 10x Additive, was added and fish were incubated for 30 min at 37 °C, protected from light. Finally, the samples were washed with 3% BSA in PBS for 5 min, and PBS for 5 min. Nuclear staining was performed with DAPI (1:500 in 3% BSA) overnight at 4 °C. Imaging was performed using a Nikon A1 confocal microscope and the images were processed with ImageJ.

### Induction of RMS Tumors in Zebrafish

Tumors were induced in zebrafish via microinjection of rag2-kRASG12D, a mutated form of kRAS that is specifically expressed in muscle progenitors, leading to the generation of RMS. The rag2-kRASG12D plasmid (a gift from the Langenau lab, Harvard Medical School, USA) was linearized using XhoI (New England Biolabs, MA, USA), as described previously.^32^ It was then purified with the NucleoSpin Gel and PCR cleanup mini kit (Macherey–Nagel GmbH & Co. KG, Germany). A concentration of 30 ng/μL of the linearized DNA was injected at the 1-cell stage in transgenic TgBAC­(pax3a:EGFP)^i150^,^38^ zebrafish, where RMS tumors were identified by the abnormal accumulation of pax3a:EGFP.^35^

### Treatment of RMS Tumors with **DBI**

**DBI** was tested on zebrafish with pax3a:EGFP RMS tumors to assess its effects in tumor regression. Tumors were imaged using a Nikon A1 confocal microscope and fish were incubated with 1 μM **DBI** for 2–3 days. Larvae were anesthetized using tricaine and **DBI** was then activated in the tumors by illuminating them with a 488 nm laser (20% intensity, 30 s). Controls included tumors treated with DMSO (0.02% v/v) and illumination, or 1 μM **DBI** without illumination. Treated larvae were kept separately, monitored, and when applicable subjected to repeated treatments. Imaging was performed using a Nikon A1 confocal microscope, and tumor areas were quantified using ImageJ.

Some larvae with tumors and healthy siblings were collected after the first treatment and subjected to TUNEL assay to detect apoptotic cells. TUNEL-positive area was quantified using ImageJ.

### Immunohistochemistry

Whole-mount immunohistochemistry was performed to study the effect of **DBI** on neuromuscular junctions. Pax3a:EGFP larvae at 1dpf were treated with **DBI** for 3 days then exposed to light or kept in the dark as controls. After treatment, larvae were incubated overnight at 28.5 °C in the dark, fixed in 4% PFA for 1 h at room temperature, and permeabilized with cold acetone for 1 h at −20 °C. Blocking was performed with a solution of 1% blocking reagent, 5% DMSO, and 4% Triton in PBT for 1 h at room temperature. Then, samples were incubated for 3 days at 4 °C with Monoclonal Anti-Acetylated Tubulin (1:100, Sigma) and for 2 days at 4 °C with goat antimouse Alexa Fluor 594 (1:500, Invitrogen) and α-Bungarotoxin Alexa Fluor 647 (1:200, Sigma) in blocking solution.

To detect G4 DNA, immunohistochemistry was performed on tissue sections. Two month-old pax3a:EGFP zebrafish with RMS tumors were fixed in 4% PFA overnight at 4 °C and stepwise incubated in 10, 20 and 30% sucrose for 12 h each at 4 °C. The samples were then mounted using OCT cryomount, frozen at −80 °C, and sectioned into 12 μm thick sections using a cryostat. Sectioned tissue was incubated with blocking solution for 1 h at RT, and with primary antibody anti-BG4 (1:100, ref. no.: Ab00174–30.126) overnight at 4 °C. After washing with PBS, the samples were incubated with rabbit anti-FLAG M2 Antibody (1:200, Cell Signaling, ref. no.:14793) for 1 h at room temperature, washed again, and incubated with goat antirabbit Alexa Fluor 633 (1:500, Invitrogen) for 1 h at room temperature. Nuclei were stained with DAPI, and samples were stored in 80% glycerol. Imaging was performed using a Nikon confocal microscope.

### BrdU Incorporation Assay

To label proliferating cells, zebrafish larvae with RMS tumors were treated with **DBI** for 3 days, illuminated, and incubated with 10 mM 5-bromo-2′-deoxyuridine (BrdU), for 24 h at 28.5 °C. Nonilluminated tumors and healthy siblings were used as controls. The larvae were fixed in 4% PFA for 2 h at room temperature, washed with PBT and treated with acetone for 1 h at −20 °C. After washing, the larvae were treated with 2 N HCl for 1 h, followed by additional washing and blocking. For BrdU detection, an Alexa Fluor 555 Mouse anti-BrdU (BD Biosciences) antibody was used at 1:20 concentration. Nuclei were stained using DAPI (Sigma). Stained embryos were stored in 80% glycerol, and images were acquired using a Nikon confocal microscope.

### **DBI** Activation on Blood and Lymph Vessels

To assess the effect of **DBI** on blood and lymphatic vessels, the Tg­(fli1a:EGFP; lyve1b:dsRED) zebrafish line (gift from the Koltowska lab) was used, with fli1a specifically expressed in blood vessels and lyve1b marking lymphatic vessels. Zebrafish at 5 dpf were incubated with 1 μM of **DBI** for 3 days and illuminated with 20% intensity of a 488 nm laser for 30 s. Control fish were not exposed to light. All samples were incubated in the dark overnight at 28.5 °C, fixed in 4% PFA for 2 h at RT, washed with PBT and stored in 80% glycerol. Images were acquired using a Nikon confocal microscope.

### Statistical Analysis

For the uptake experiments, fluorescence was measured in the following samples: DMSO 2 dpf (*n* = 5), **DBI** 2 dpf (*n* = 5), DMSO 7 dpf (*n* = 10), **DBI** 7 dpf (*n* = 10). Two-way ANOVA was performed with Bonferroni multiple comparisons test.

The tumor areas, expressed as a percentage of the pretreatment size, were measured at 4–5 days post treatment in four conditions: **DBI** with light (treatment, *n* = 10), **DBI** without light (control, *n* = 8), DMSO with light (control, *n* = 7), and DMSO without light (control, *n* = 6). A one-way ANOVA was conducted to determine if mean tumor area measurements differed significantly among the four groups. A posthoc Tukey’s multiple comparisons test was then used to identify specific group pairs with significant differences.

For the apoptosis detection in larvae with tumors and healthy siblings, TUNEL positive area was measured in the following samples: **DBI** – light healthy (*n* = 5), **DBI** – light tumor (*n* = 10), **DBI** + light healthy (*n* = 20), **DBI** + light tumor (*n* = 12). Two-way ANOVA was performed with Bonferroni multiple comparisons test.

Statistical significance was indicated as follows: *p* < 0.05 (*), *p* < 0.01 (**), *p* < 0.001 (***), *p* < 0.0001 (****). Statistical analyses and graphs were created using BioRender.

## Discussion

RMS is a rare but aggressive soft tissue sarcoma that predominantly affects children and teenagers.^39^ This cancer presents significant treatment challenges, including a high recurrence rate and the limited efficacy of conventional therapies, particularly in advanced or metastatic cases. Given these limitations, PDT offers a promising approach, as it targets tumor cells while minimizing damage to surrounding healthy tissue. Although PDT has demonstrated efficacy in treating various cancers,^3–6^ its application in RMS remains underexplored, with only a limited number of in vitro studies available.^7,8,40^ While these studies have shown encouraging results, translating these findings into in vivo models remain a critical challenge. Zebrafish models provide an advantageous platform for addressing this gap, as their small, transparent larvae allow for in vivo testing compound toxicity and efficacy. These characteristics, combined with the optical clarity of tissues, make zebrafish ideal for real-time visualization of tumor growth and response to light-induced treatments using fluorescence microscopy. Additionally, zebrafish are suitable for studying RMS due to their genetic similarities to human cancer biology.^27,33^

In our study, we tested PDT in zebrafish with kRAS-induced RMS tumors using the fluorescent molecule **DBI** as a photosensitizer. We evaluated the uptake and activation of **DBI**, its apoptotic effects, and its impact on tumor reduction in zebrafish larvae with RMS. Apoptosis was significantly increased only in embryos treated with **DBI** in the presence of blue light. These findings confirm that **DBI**-induced toxicity requires both the presence of the photosensitizer and light exposure, demonstrating that **DBI** effectively induces targeted DNA damage upon light activation. We found that RMS tumor tissue was significantly more affected by the light activated **DBI** than healthy tissue, indicating that **DBI** treatment is efficient in treating RMS in the context of our model.

Our data also indicate that larger vessels are less affected by **DBI** treatment, which could argue for increased risk of relapse if the tumor cells continue to have a flow of nutrients post treatment. Importantly, we did not observe an increased proliferation in the treated area in RMS compared to healthy tissue. We also did not observe a quick recovery of tumor tissue, as all examined larvae treated with **DBI** displayed a reduced tumor size throughout the experimental period. The severe damage observed in nerves and neuromuscular junctions following **DBI** treatment suggests that tumor innervation may be particularly sensitive to ROS generation. Treating cancers by targeting tumor innervation is also an increasingly popular approach.^41^ Furthermore, analysis of G4 content using the BG4 antibody revealed significant G4 accumulation in RMS tumors compared to surrounding healthy tissues, suggesting their elevated G4 levels may underlie the increased sensitivity to **DBI** treatment, consistent with the role of G4s as a promising cancer target.^16,17,42^

Our RMS zebrafish model allowed us to visualize these changes in vivo, providing real-time insights into the effects of PDT on tumor cells. One of the main advantages of PDT is the ability to target tumors very precisely, minimizing damage to surrounding tissues. However, limitations exist in this type of therapy, such as difficulties with light penetration in larger tumors and ensuring effective uptake of photosensitizers by cancer cells. In larger animals, achieving deep light penetration into the tumor tissue is more challenging and requires specific adjustments for each type of cancer, photosensitizing compound and irradiation wavelength.^43^ To optimize PDT for RMS, future research could involve refining photosensitizer and light delivery methods, as well as exploring PDT in combination with other treatments to enhance efficacy. Zebrafish studies can help screen these new approaches rapidly, bridging the gap between initial research and mammalian trials.

In summary, this study evaluated PDT using the promising light-inducible molecule **DBI** in a zebrafish model of kRAS-induced RMS. The results demonstrate that PDT with such heavy-atom-free PS is a minimally invasive treatment capable of effectively inducing apoptosis and RMS tumor cell death exclusively in targeted areas, leading to reduced tumor size. These findings confirm the high potential of the **DBI** as a promising candidate molecule for future RMS therapies.

## Supplementary Material



## Notes

Additional experimental results: BrdU incorporation in Pax3a:EGFP zebrafish after **DBI** treatment and effect of **DBI** on blood and lymph vessels (PDF)
