# Supplementary material for: Photodynamic Therapy Using a Heavy-Atom-Free G‑Quadruplex-Targeted Photosensitizer to Efficiently Regress Rhabdomyosarcoma Tumors In Vivo
Source: ACS Pharmacol Transl Sci. 2025 Apr 16;8(8):2482–92. doi: 10.1021/acsptsci.5c00061 (PMC12340618; doi:10.1021/acsptsci.5c00061)
Supplement: Supplementary file 1 [file pt5c00061_si_001.pdf]

Supporting Information

Photodynamic Therapy using a Heavy-atom-free G-quadruplex-Targeted Photosensitizer to Efficiently Regress Rhabdomyosarcoma Tumors In Vivo

Eva Rodriguez-Marquez[1], Hanna Nord[1], Darío Puchán Sánchez[2], Ahmad Kassem[2], José María Andrés Castán[2], Marco Deiana[3], Clement Cabanetos[2], Nasim Sabouri[4], Jonas von Hofsten[1]*

1. Department of Medical and Translational Biology, Umeå University, 90187 Umeå, Sweden
2. Univ Angers, CNRS, MOLTECH-ANJOU, SFR MATRIX, F-49000 Angers, France
3. Institute of Advanced Materials, Faculty of Chemistry, Wrocław University of Science and Technology, 50-370 Wrocław, Poland
4. Department of Medical Biochemistry and Biophysics, Umeå University, 90187 Umeå, Sweden
* Corresponding author: jonas.von.hofsten@umu.se

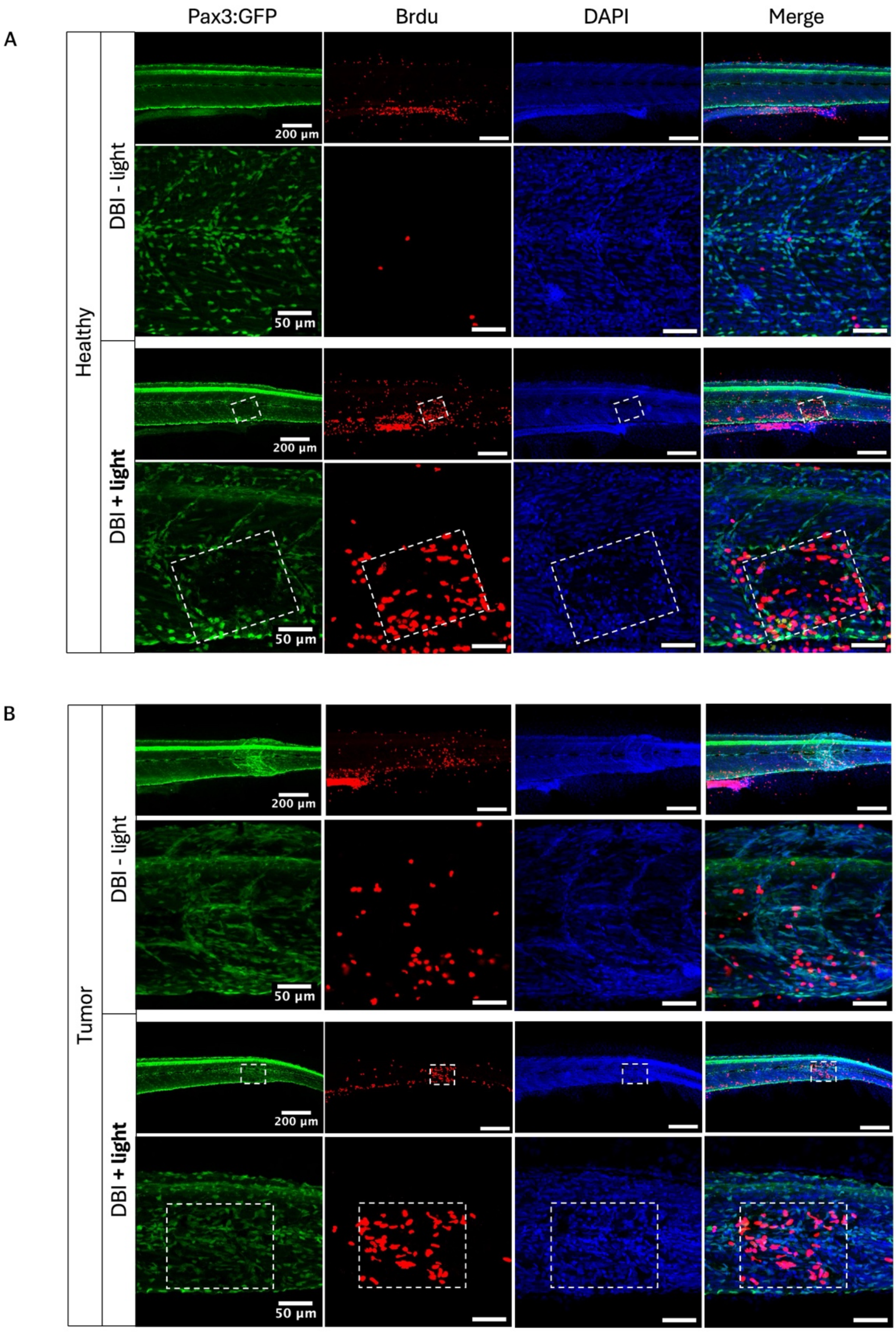

Figure S1: BrdU incorporation in Pax3a:EGFP zebrafish after DBI treatment. (A) *17dpf Pax3a:EGFP zebrafish without tumors treated with DBI, followed or not by illumination.* ***(B)*** *Sibling zebrafish with rhabdomyosarcoma tumors treated with DBI, followed or not by illumination. Dashed squares indicate the illuminated areas. BrdU is stained in red and nuclei were counterstained with DAPI (blue).*

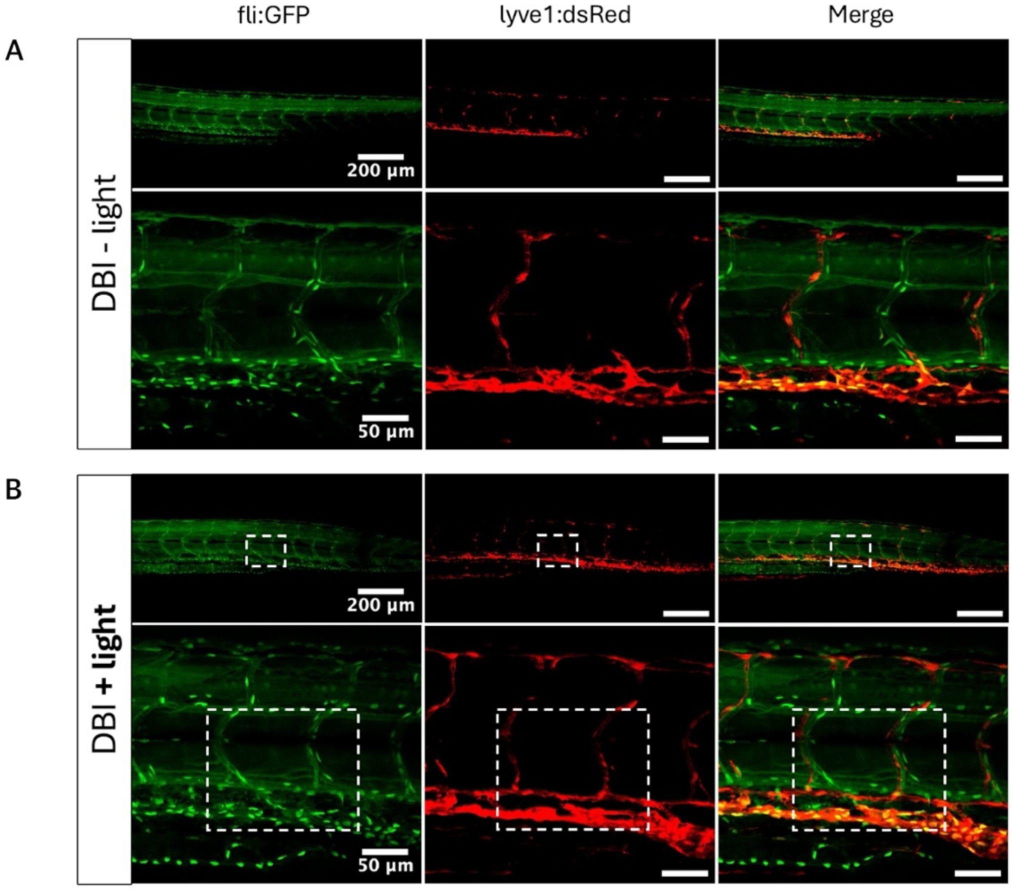

Figure S2: Effect of DBI on blood and lymph vessels. (A) *Tg(fli1:EGFP; lyve1:dsRED) zebrafish line treated with DBI without light.* ***(B)*** *Tg(fli1:EGFP; lyve1:dsRED) zebrafish line treated with DBI and light. Dashed squares indicate the illuminated areas. Fli1 is expressed in blood vessels (green) and lyve1 marks lymphatic vessels (red).*
